# Patterns of at-sea behaviour at a hybrid zone between two threatened seabirds

**DOI:** 10.1038/s41598-019-51188-8

**Published:** 2019-10-11

**Authors:** Rhiannon E. Austin, Russell B. Wynn, Stephen C. Votier, Clive Trueman, Miguel McMinn, Ana Rodríguez, Lavinia Suberg, Louise Maurice, Jason Newton, Meritxell Genovart, Clara Péron, David Grémillet, Tim Guilford

**Affiliations:** 1 National Oceanography Centre - Southampton, European Way, Southampton, SO14 3ZH UK; 2 Environment and Sustainability Institute, University of Exeter, Penryn Campus, Penryn, Cornwall TR10 9FE UK; 3 Ocean and Earth Sciences, University of Southampton Waterfront Campus, Southampton, SO14 3ZH UK; 4 Grupo Biogeografía, geodinámica y sedimentación del Mediterráneo occidental (BIOGEOMED), Universitat de les Illes Balears, Cra. de Valledemossa, km 7.5, E07122 Palma, Balearic Islands Spain; 5 British Geological Survey, Natural Environment Research Council, Benson Lane, Crowmarsh Gifford, Oxfordshire OX10 8BB UK; 6 NERC Life Sciences Mass Spectrometry Facility, Scottish Universities Environmental Research Centre, East Kilbride, Scotland G75 0QF UK; 7 IMEDEA (CSIC-UIB), Miquel Marquès 21, 07190 Esporles, Mallorca Spain; 8 CEAB (CSIC), Accés Cala Sant Francesc 14, 17300 Blanes, Girona, Catalonia Spain; 9 Centre d’Ecologie Fonctionnelle et Evolutive, UMR 5175, CNRS – Université de Montpellier - Université Paul-Valéry Montpellier - EPHE, Montpellier, France; 10 Muséum national d’Histoire naturelle, Département Adaptations du vivant, UMR 7208 BOREA (MNHN, CNRS, IRD, Sorbonne Université, UCB, UA), CP 26, 43 rue Cuvier, 75231 Paris cedex 05, France; 11 FitzPatrick Institute, DST-NRF Centre of Excellence at the University of Cape Town, Rondebosch, 7701 South Africa; 12 Department of Zoology, University of Oxford, 11a Mansfield Road, Oxford, OX1 3SZ UK; 13 Present Address: School of Environmental Sciences, University of Liverpool, Nicholson Building, Brownlow Street, Liverpool, L69 3GP UK

**Keywords:** Animal migration, Behavioural ecology, Evolutionary ecology, Stable isotope analysis, Marine biology

## Abstract

Patterns of behavioural variation and migratory connectivity are important characteristics of populations, particularly at the edges of species distributions, where processes involved in influencing evolutionary trajectories, such as divergence, mutual persistence, and natural hybridization, can occur. Here, we focused on two closely related seabird species that breed in the Mediterranean: Balearic shearwaters (*Puffinus mauretanicus*) and Yelkouan shearwaters (*Puffinus yelkouan*). Genetic and phenotypic evidence of hybridization between the two species on Menorca (the eastern and westernmost island in the breeding ranges of the two shearwaters, respectively) has provided important insights into relationships between these recently diverged species. Nevertheless, levels of behavioural and ecological differentiation amongst these populations remain largely unknown. Using geolocation and stable isotopes, we compared the at-sea movement behaviour of birds from the Menorcan ‘hybrid’ population with the nearest neighbouring populations of Balearic and Yelkouan shearwaters. The Menorcan population displayed a suite of behavioural features intermediate to those seen in the two species (including migration strategies, breeding season movements and limited data on phenology). Our findings provide new evidence to support suggestions that the Menorcan population is admixed, and indicate a role of non-breeding behaviours in the evolutionary trajectories of *Puffinus* shearwaters in the Mediterranean.

## Introduction

Behavioural and ecological processes can be central to understanding patterns of evolutionary differentiation amongst animal populations^1–4^, particularly in wide-ranging marine vertebrates, such as seabirds, unconstrained by the physical barriers to gene flow that promote species divergence in other taxa^5^. Factors such as non-breeding segregation^5^, habitat specialization^6,7^, natal philopatry^8^ and mate preference^9^ can all play a role in promoting genetic isolation in this group. Furthermore, the pattern of intermediate behavioural phenotypes in a hybrid zone can help indicate the potential resilience or vulnerability of closely related species to admixing, as a result of secondary contact. Understanding variation in movement behaviour, migratory connectivity and habitat choice is therefore particularly important for identifying the evolutionary relationships amongst marine vertebrates^10^, as well as to better inform their conservation^11^. Here we explore the spatio-temporal patterns of seasonal movement behaviour and habitat use in two closely related shearwater species from the Mediterranean Sea, the Balearic shearwater *Puffinus mauretanicus* and the Yelkouan shearwater *P. yelkouan*, at breeding colonies close to a presumed contact zone, and in a third, probable hybrid population breeding in that zone.

The present taxonomic status of *Puffinus* shearwaters in the Mediterranean follows successive taxonomic revisions, initially separating these two species from the Manx shearwater *P. puffinus*^12,13^, and later from each other^14–16^. The Mediterranean *Puffinus* species are believed to have diverged from a common North Atlantic ancestor ~1 million years ago^17^, after the reopening of the Strait of Gibraltar^12,15,18,19^. In recent years, the taxonomic relationship between Balearic and Yelkouan shearwaters has been the subject of considerable study^17,20–22^. This is partly because of evidence for widespread population declines^23^ (they are currently listed as Critically Endangered and Vulnerable, respectively^24,25^), but also owing to discovery of a ‘hybrid’ population at the easternmost extent of the Balearic shearwater’s breeding range (also believed to be in decline)^17^. Although technically classified as a Balearic shearwater, genetic analyses of mitochondrial DNA (mtDNA) have revealed the presence of both Balearic and Yelkouan haplotypes in the *Puffinus* population on the island of Menorca, providing evidence for natural hybridization between the two species^17,20,22^. This process has both evolutionary and conservation significance as hybridization could influence, or be accentuated by, ongoing declines of one or both species^21,26^.

Phenotypic differences between shearwaters from Menorca (hereafter referred to as ‘Menorcan shearwaters’), and Balearic shearwaters elsewhere in archipelago have also been detected, including those related to morphology^21,22,27–29^, plumage coloration^21,22^, vocalisations^30,31^ and breeding phenology^30,32^. Together, these differences have led to uncertainties surrounding the status of the Menorcan population. However, other behavioural and ecological attributes of shearwaters on this island remain largely unknown. While recent tracking studies have provided insights into the year-round at-sea distributions of Balearic shearwaters from western colonies^33,34^ and Yelkouan shearwaters from multiple colonies^35,36^, there is a lack of comparable at-sea information for Menorcan shearwaters (although see^29^), owing partly to the low accessibility of nesting birds on Menorca (McMinn, personal observation).

In this study, we focus on behavioural and ecological differentiation between the Menorcan population, a Balearic shearwater population from neighbouring Mallorca Island, and one of the nearest Yelkouan shearwater populations on the French Mediterranean coast (Port Cros and Porquerolles Islands)^35^, to improve understanding of the evolutionary processes acting on these species. We present the first year-round tracking data of Menorcan shearwaters and combine this with stable isotope analyses to investigate migratory behaviour, spatial ecology and, briefly, phenology of these enigmatic birds. One of the key aims of this study was to test whether post-breeding Menorcan birds migrate into northeast Atlantic waters as Balearic shearwaters from western colonies do^33,34^, or remain in the Mediterranean in a similar fashion to Yelkouan shearwaters^35–37^. We compared new contemporaneous data from Balearic shearwaters on Mallorca and from Menorcan shearwaters, to existing published data from Yelkouan shearwaters^35^ that were reanalysed using consistent methodologies. Our results provide new behavioural and ecological insights into Menorcan shearwaters of relevance to this population’s taxonomic status, and highlight the potential role of at-sea behaviours in the differentiation of closely related seabird populations.

## Results

### Inter-population differences in at-sea movements

Thirteen out of 25 GLS devices were recovered from birds on Menorca, yielding 10 tracks that encompassed non-breeding periods between 2011 and 2013 (*n*, 2011 = 2, 2012 = 2, 2013 = 6), and 3 devices that failed to provide data. A total of 52 comparison tracks (and simultaneously collected salt-water immersion data) were collected from birds recovered on Mallorca (*n*, 2011 = 16, 2012 = 16, 2013 = 20), and 34 were obtained from Yelkouan shearwaters from France (*n*, 2011 = 15, 2012 = 19^35^).

**Figure 1:**
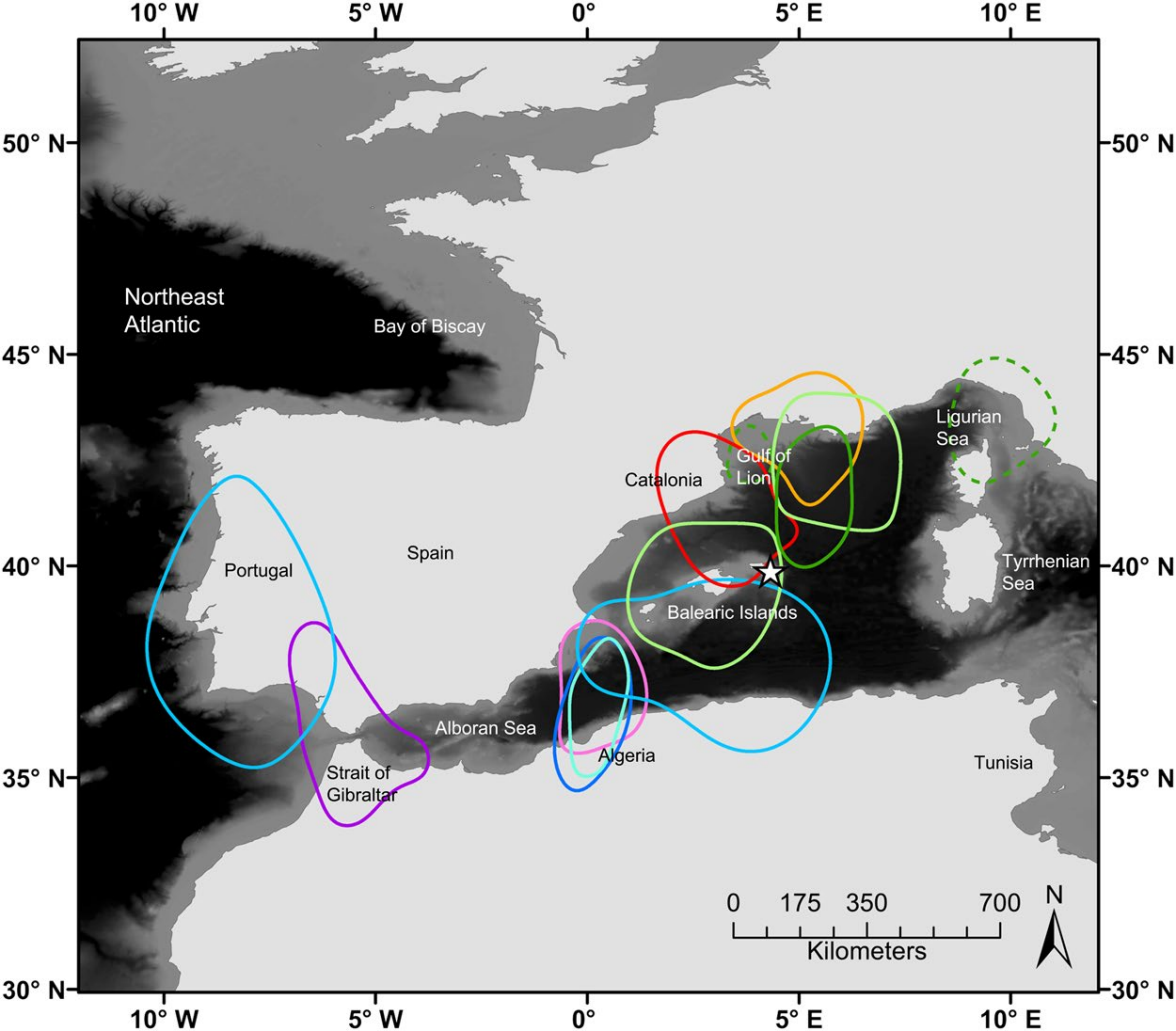
Non-breeding distributions of individual shearwaters from Menorca. 50% kernel density distributions of Menorcan shearwaters tracked with geolocators from La Mola, Menorca during non-breeding periods between 2011 and 2013 are shown. Kernel density estimates of individual birds are provided in different colours (*n* = 10; green = 2011, red/orange = 2012, blue/purple = 2013, dotted green = 2013 track from individual shown in the same colour in 2011). KDE bandwidth selector = plug-in; non-breeding period = time between the last night visit to the colony before departure for migration and first night visit to the colony after return; star = colony location; bathymetry = GEBCO 30-arc second data.

Eight tracked Menorcan birds (80%) spent the non-breeding season in the western Mediterranean (Fig. 1). The core distribution of individuals within this region was highly variable, spanning areas up to 1400 km from the Ligurian Sea to the Alboran Sea. One individual was tracked in two separate years and visited non-breeding areas between the Gulf of Lion and Ligurian Sea in both (Fig. 1). The other two Menorcan birds were located further west during the non-breeding season, their core distributions extending into northeast Atlantic waters between the Straits of Gibraltar and central Portugal (Fig. 1).

**Figure 2:**
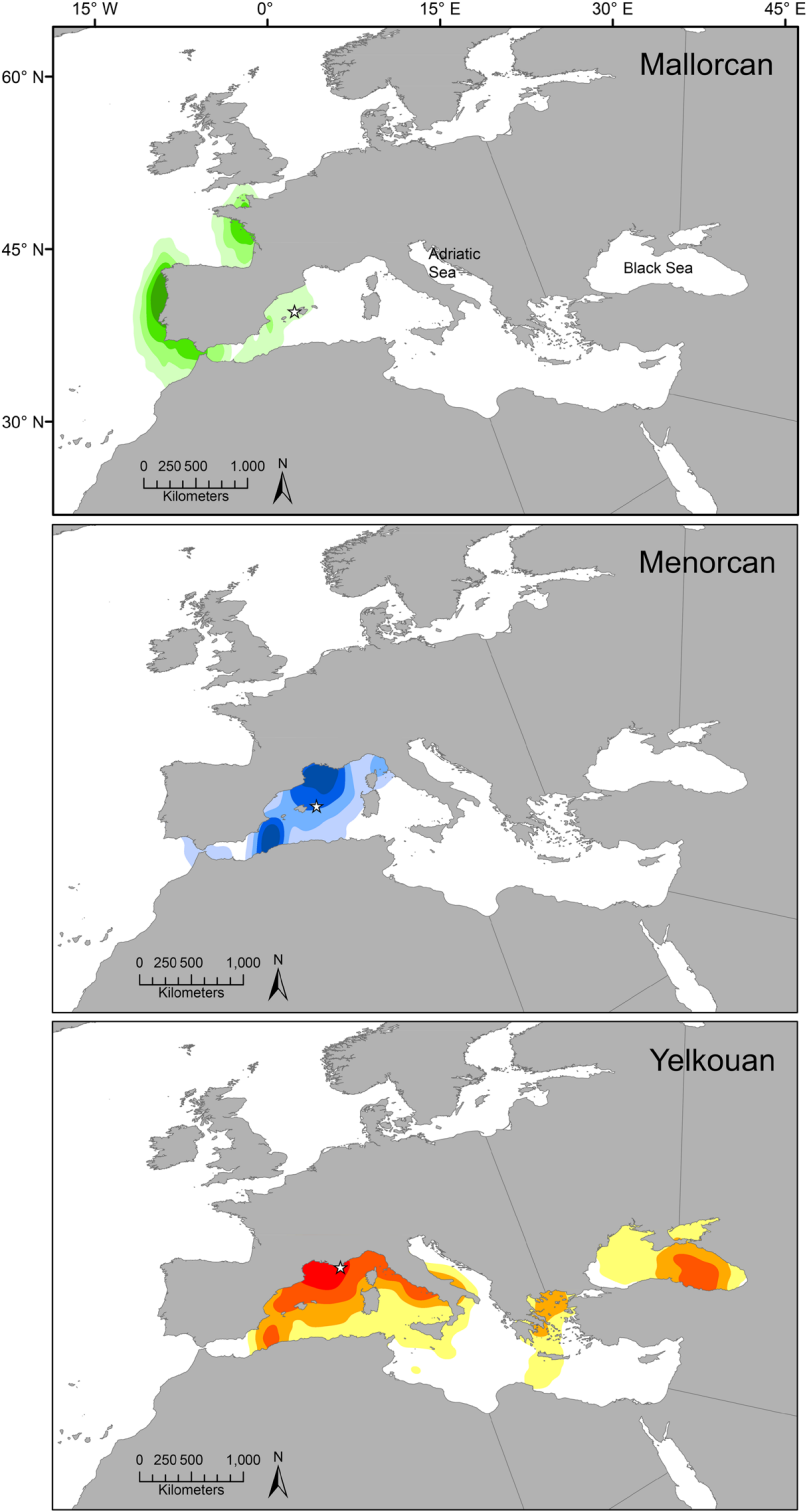
Comparison of the non-breeding distributions of Mallorcan, Menorcan and Yelkouan shearwater populations. Kernel density estimates of geolocator-tracked Balearic shearwaters from Sa Cella, Mallorca (**a**), Menorcan shearwaters from La Mola, Menorca (**b**), and Yelkouan shearwaters from a population in the Hyères Archipelago, French Mediterranean (**c**; data source^35^), during non-breeding periods between 2011 (*n*, Mallorca = 16; Menorca = 2; France = 15) and 2012 (*n*, Mallorca = 16; Menorca = 2; France = 19) are shown. Bandwidth selector = plug-in; 25%, 50%, 70% and 90% kernel density contours are given. Stars = colony locations. Maps created in ArcGIS version 10.0 (ESRI, USA; http://desktop.arcgis.com/en/arcmap/). See supplementary information for separate kernels by year.

**Figure 3:**
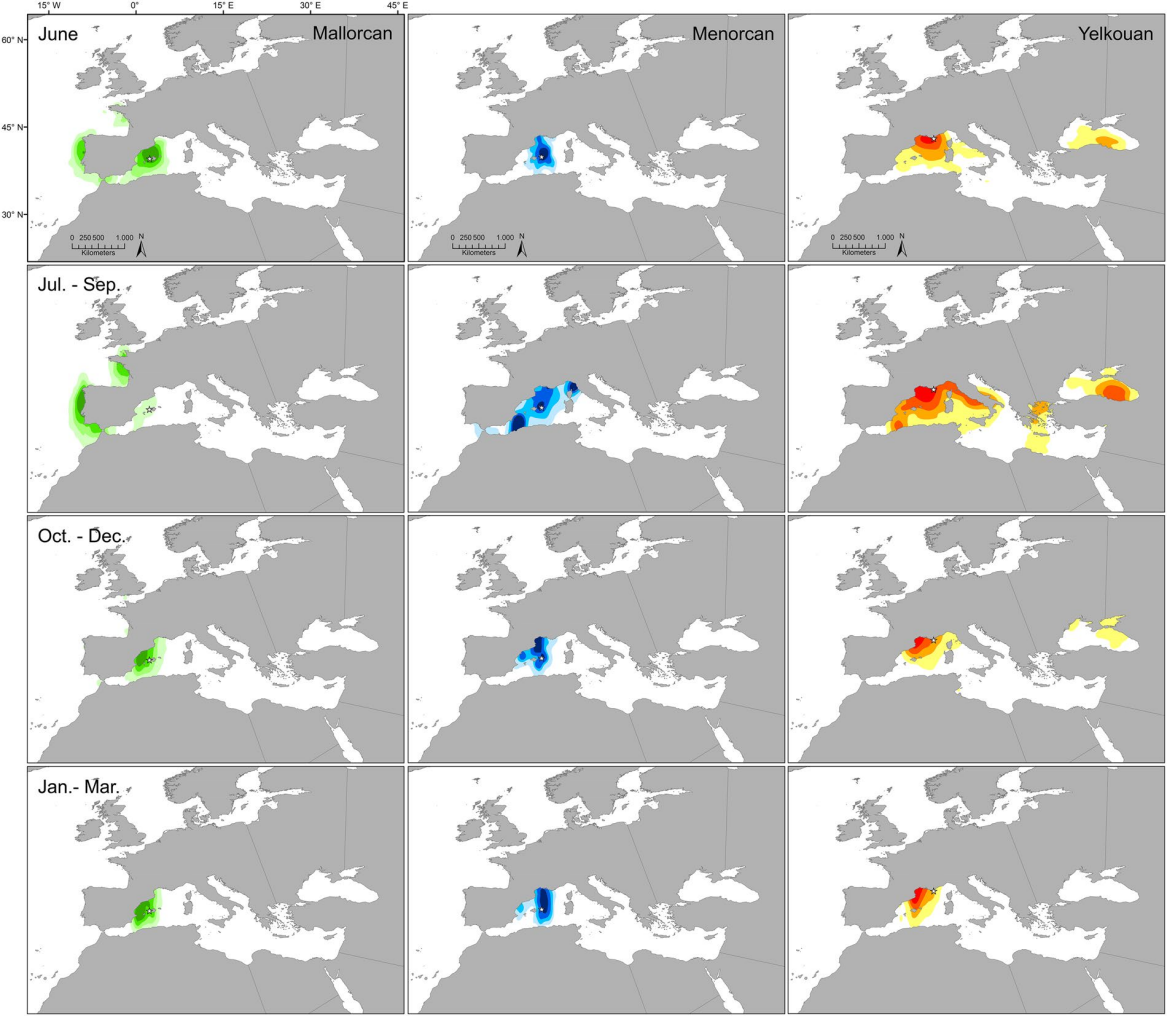
Distributions of Mallorcan, Menorcan and Yelkouan shearwaters throughout the year. Kernel density estimates of shearwaters tracked with geolocators from Sa Cella, Mallorca (*n* = 52), La Mola, Menorca (*n* = 10) and the Hyères Archipelago, France (*n* = 34) are shown for June, July – September, October – December and January – March (2011–2014). 25%, 50%, 70% and 90% contours are given. Stars = colony locations. Maps created in ArcGIS version 10.3 (ESRI, USA; http://desktop.arcgis.com/en/arcmap/).

There were notable differences in the non-breeding distributions of birds from the three colonies. Mallorcan shearwaters predominantly migrated to, and spent the bulk of the non-breeding period in, Atlantic waters off western Iberia and northwest France (84% of tracked birds). In comparison, Menorcan shearwaters showed movements largely restricted to the western Mediterranean, whilst tracked Yelkouan shearwaters combined movements in the western Mediterranean with some migration further eastwards into the Black Sea (Figs 2 and 3; Supplementary Fig. S1). Between October and June (the main pre-breeding and breeding seasons), the core at-sea Mediterranean distribution of the Menorcan population fell further east than that of Mallorcan birds, showing more similarity to that of Yelkouan shearwaters (Fig. 3).

### Isotopic variation between non-breeding regions

**Figure 4:**
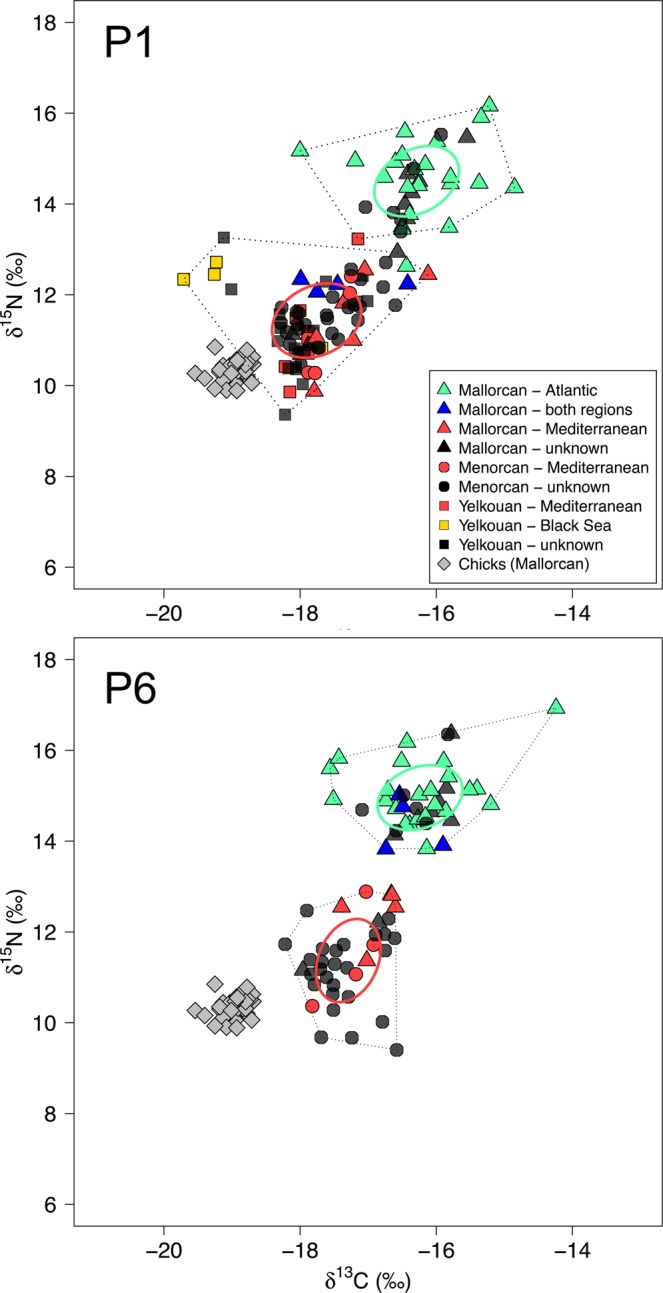
Comparison of feather stable isotope values from Mallorcan, Menorcan and Yelkouan shearwater populations. Carbon and nitrogen stable isotope values in primary feathers of geolocator-tracked adult Balearic shearwaters from Mallorca (coloured triangles, P1 and P6), Menorcan shearwaters from Menorca (coloured circles, P1 and P6), and Yelkouan shearwaters from the Hyères Archipelago, France (coloured squares, P1 only; data source^35^) are shown. Feather isotope values relate to non-breeding periods in 2011 (BS and YS) and 2012 (BS only). Black points represent untracked birds from the three colonies. Isotope values for BS chick down collected from the Mallorcan colony in June 2013 are shown to provide a Mediterranean reference (diamonds). Solid lines represent standard ellipse areas (corrected for small sample sizes: SEAc) for two groups defined by hierarchical clustering analysis. Dotted lines show convex hulls.

*δ*^15^N and *δ*^13^C values of feathers grown during the non-breeding period differed significantly between the three populations (Table 1). Hierarchical clustering analysis identified two distinct isotopic groups from *δ*^15^N and *δ*^13^C data (Fig. 4; Supplementary Fig. S2). Isotopic values in P6 feathers sampled from tracked Balearic shearwaters that spent the non-breeding period in Mediterranean and Atlantic waters differentiated into separate clusters. A similar pattern was observed for P1, with most Mallorcan and Yelkouan shearwaters falling into the ‘Atlantic’ and ‘Mediterranean’ clusters respectively, and Menorcan shearwaters spanning both although predominantly falling within the Mediterranean cluster.

**Table 1:** Comparison of feather stable isotope values between Mallorcan, Menorcan and Yelkouan shearwaters. Mean (±SD) *δ*^15^N and *δ*^13^C values of primary feathers (P1 and/or P6) from adult Balearic shearwaters from Mallorca, Menorcan shearwaters from Mallorca, and Yelkouan shearwaters from two colonies in the French Mediterranean, grown during non-breeding seasons in 2010, 2011 and/or 2012 are shown. Numbers in square parentheses represent sample sizes. Chi-square values and *p*-values summarize likelihood ratio tests used to compare full linear mixed-effects models (with ‘colony’/’year’ as a fixed effect and ‘individual’ as a random intercept term) with models containing no fixed effect. No significant differences in isotope ratios were found between years, with the exception of *δ*^13^C for P1.

Isotope ratio	Year	P1			P6	
		Mallorca	Menorca	France	Mallorca	Menorca
*δ*^15^N (‰)	2010	—	—	11.2 (±0.7) [35]	—	—
	2011	13.7 (±1.6) [23]	12.1 (±1.4) [20]	11.3 (±1.0) [33]	14.5 (±1.3)	11.8 (±1.5)
	2012	13.7 (±1.6) [18]	11.8 (±0.8) [16]	—	14.5 (±1.3)	11.8 (±1.8)
	LRT	χ^2^_2_ = 44.850 P < 0.001^a^			χ^2^_1_ = 37.459 P < 0.001^b^	
*δ*^13^C (‰)	2010	—	—	−17.7 (±0.6) [35]	—	—
	2011	−16.9 (±0.7) [23]	−17.5 (±0.7) [20]	−18.1 (±0.6) [33]	−16.5 (±0.7)	−17.4 (±0.6)
	2012	−16.1 (±0.8) [18]	−17.2 (±0.4) [16]	—	−16.1 (±0.7)	−16.9 (±0.4)
	LRT	χ^2^_2_ = 74.222 P < 0.001^b^			χ^2^_1_ = 33.885 P < 0.001^b^	

^a^Significant differences lay between Mallorcan shearwaters and the other two populations only; ^b^Significant differences lay between all pairwise comparisons (Tukey’s posthoc tests, P < 0.001).

The discriminant function analysis (DFA) on P6 isotopes, which represents diet during the centre of the non-breeding period, correctly assigned all 26 tracked birds in the training data to their non-breeding area (100% accuracy; Wilks’ Lambda 0.221, *F* = 84.725, χ^2^ = 34.755, P < 0.001). A leave-one-out cross-validation of the training data also correctly classified 100% of birds. 26 of 32 (81%) untracked Menorcan birds were then predicted by the discriminant function to have spent the non-breeding season in the Mediterranean, and all predictions agreed with clusters defined by hierarchical cluster analysis (Fig. 4; Supplementary Fig. S2). As no matching P6 data were available for Yelkouan shearwaters, a DFA was also run on P1 isotope values from the three sampled populations, representative of diet during the beginning of the non-breeding period. This analysis correctly assigned 32 of 36 birds to their non-breeding area in both original and cross-validated group cases (84% accuracy; Wilks’ Lambda 0.400, *F* = 45.388, χ^2^ = 32.044, P < 0.001). Five of 6 incorrectly assigned birds were from Sa Cella, Mallorca. This lower accuracy for P1 likely reflects uncertainty in the region of P1 growth over a period of migration at the beginning of the breeding season.

The obtained discriminant functions were:

$${D}_{p1}=0.666\times {\delta }^{15}N+0.538\times {\delta }^{13}C+0.828$$

$${D}_{p6}=1.235\times {\delta }^{15}N-0.033\times {\delta }^{13}C-17.995$$

Fisher’s classification functions:

$${D}_{p1}(Mediterranean)=14.743\times {\delta }^{15}N-36.551\times {\delta }^{13}C-412.491$$

$${D}_{p1}(Atlantic)=16.331\times {\delta }^{15}N-35.270\times {\delta }^{13}C-410.518$$

$${D}_{p6}(Mediterranean)=35.138\times {\delta }^{15}N-55.545\times {\delta }^{13}C-684.788$$

$${D}_{p6}(Atlantic)=39.971\times {\delta }^{15}N-55.674\times {\delta }^{13}C-751.429$$

**Figure 5:**
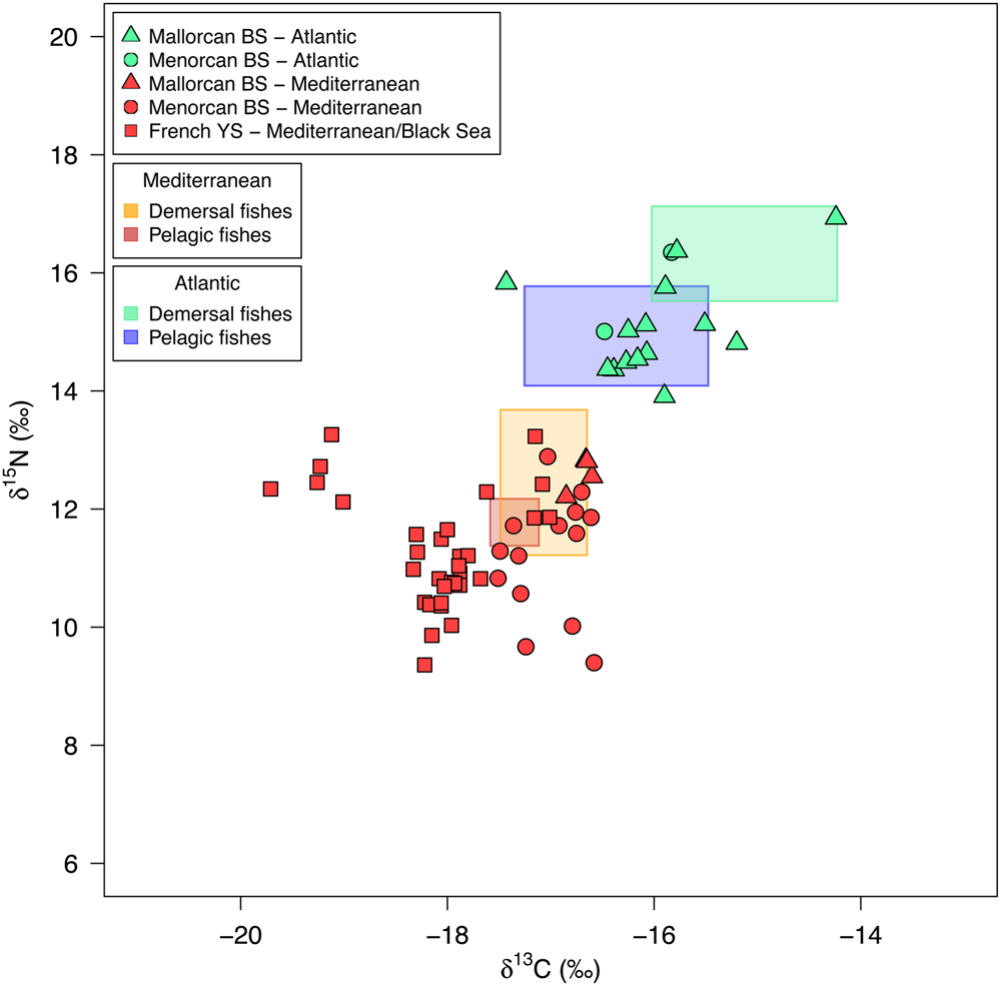
Stable isotope values from Mallorcan, Menorcan and Yelkouan shearwater feathers and potential prey. Carbon and nitrogen stable isotope values in P6 feathers of shearwaters from Menorcan and Mallorcan colonies grown during summer 2012, and P1 feathers of Yelkouan shearwaters from the Hyères Archipelago, France grown during 2011, are shown. Birds are coloured by the non-breeding area that they were tracked to or were predicted to have used during the non-breeding season based on a discriminant function analysis (green = Atlantic, red = Mediterranean/Black Sea). Coloured boxes indicate the fractionation-corrected isotope space (mean ± SD) of pelagic and demersal fishes (<20 cm total length) collected from coastal Atlantic (*n* = 89) and Mediterranean (*n* = 69) waters during spring – summer 2012.

The isotope values of feathers grown during the non-breeding period (P6 – Balearic, P1 – Yelkouan) overlapped with fractionation-corrected isotope space of both pelagic and demersal fishes in Mediterranean and Atlantic regions sampled over similar timescales (Fig. 5 ; Supplementary Table S1). There was little overlap in the isotope niche space of fishes from the two regions, and most birds occupied isotope prey space delimited by fish samples collected in the region that they were either tracked to or were predicted (by DFA) to have used (Fig. 5). The *δ*^15^N values of some Mediterranean-assigned birds were lower than the sampled prey space, potentially indicating some exploitation of lower trophic level prey, or migratory movements into food webs further east than our sampling range.

### Phenology and plumage variation

Phenological attributes were extracted from immersion data collected for all birds tracked with geolocators and used in spatial analyses of migration behaviour, plus an additional two Yelkouan shearwaters for which only partial light data were recovered. Geolocator data indicated that the timing of key annual cycle events for tracked individuals from all three populations was variable (see Supplementary Fig. S3 and Table S2). Small sample sizes at the Menorcan colony prevented statistical comparisons of phenology within year. Nevertheless, in our combined data, we found initial indications for differences in the timings of when birds began visiting their colony during the day (Likelihood ratio test: χ^2^_2_ = 21.705, P < 0.001), in laying date (Likelihood ratio test: χ^2^_2_ = 24.730, P < 0.001), and in the last day visit to the colony (signifying the hatching date, as birds cease daytime visits shortly after this date; Likelihood ratio test: χ^2^_2_ 23.243, P < 0.001). Mallorcan shearwaters showed earlier onsets of these events, on average, than either one or both of the other populations (see Supplementary Fig. S3 and Table S2). This was the case even after removal of failed breeders in the Mallorcan data (the only population that breeding success data were available for). These patterns are broadly consistent with those observed in spatial data. Nonetheless, replication is limited here and further investigation of phenological differences is required.

Eight out of 10 tracked birds from Menorca had white underparts consistent with plumage colouration of Yelkouan shearwaters, all but one of which remained in the Mediterranean (see Supplementary Fig. S4 for examples of plumage differentiation between the two study populations). The other two tracked Menorcan shearwaters had mostly white plumage with some limited mottling on the undertail feathers and beginnings of a pectoral collar (score 2 on a scale of 1–5, following^21^). One of the two birds that were tracked into the northeast Atlantic had yelkouan-type plumage colouration.

## Discussion

Here, we report for the first time the non-breeding movements, year-round distribution and (initial data on) breeding phenology of Menorcan shearwaters – a presumed hybrid population between Balearic and Yelkouan shearwaters – providing a comparison of these characteristics with those in parent species breeding at two of the closest colonies. Our study indicates that this population displays a distinct suite of behavioural traits largely *intermediate* to those of Balearic and Yelkouan shearwaters, of relevance to our understanding of the evolution of *Puffinus* shearwaters in the Mediterranean.

We found clear differences in the migratory behaviour of the three study populations. Tracking and isotope data both suggest that the majority of Menorcan birds (80% tracked individuals and 81% isotope-sampled individuals) remain resident in the western Mediterranean post-breeding, while all Balearic shearwaters from Mallorca (the nearest neighbouring colony, 171 km away) engaged in westward movements, most extending into the northeast Atlantic for at least part or all of the non-breeding period (98% of tracked birds, 82% of isotope-sampled birds), indicating the presence of different controls on migration. The two Menorcan birds that did exit the Mediterranean remained predominantly in southwest Iberian waters, showing little overlap with frequented non-breeding areas of Balearic shearwaters off northwest Iberia and northwest France^33,34,38^ (Fig. 2). Conversely, whilst the non-breeding movements exhibited by the Menorcan population in the western Mediterranean show partial similarity to the migration behaviour of tracked Yelkouan populations in recent years, we found no evidence of movements further to the east which are shown by Yelkouans. A re-analysis of tracking data from Yelkouan shearwaters in colonies on the Mediterranean French coast^35^ (Figs 2 and 3; Supplementary Fig. S1), in addition to published data from Malta^36^, indicates that this species remains in the Mediterranean or Black Sea post-breeding, verifying that its migratory behaviour is also distinctly different to that of Mallorcan Balearic shearwaters, but also indicating that Menorcan birds may be intermediate in their movements between the two parent species. There is additional evidence for colony-based differences in Yelkouan shearwater movements, with a large proportion of birds from French populations engaging in movements further west than birds breeding on Malta, which either remain resident in central regions or migrate to the eastern Mediterranean^35,36^. These findings are consistent with the limited visual sightings records of Yelkouan shearwaters in Atlantic waters (particularly north of the Iberian coast^32^), coupled with suggestions that Menorcan birds are inseparable at sea from Yelkouan shearwaters^29,32^. A comparison of isotopic data from our three study populations further supports the tracking data (Figs 4 and 5; Supplementary Fig. S2 and Table S1). In light of these observed differences in migratory behaviour, we suggest that there may be a gradient of migration strategies in *Puffinus* populations across the Mediterranean. The hybrid zone on Menorca may in fact fall at the centre of this gradient, which could correspond to a form of migratory divide whereby populations either side show divergent migratory directions, as seen in some terrestrial species^2,39,40^. Nevertheless, further simultaneous tracking of a wider range of populations covering the full extent of the species’ breeding ranges would be needed to explore this hypothesis thoroughly.

The proportion of Menorcan shearwaters predicted and observed to remain in the Mediterranean by feather isotope compositions and tracking data, respectively, was highly consistent (~0.8 with both methods). Feather isotope compositions corresponded to clear *δ*^15^N and *δ*^13^C differences between Mediterranean and Atlantic systems, seen in our reference prey isotope data, and more widely at lower trophic levels^41–45^. Moreover, our findings are consistent with existing interpolated and modelled isoscapes of Atlantic and Mediterranean baselines, based on dissolved inorganic carbon, phytoplankton and zooplankton^46–48^. Observed regional-scale differences in feather isotopes are therefore likely to reflect spatial variability in baseline community biochemistry and sources of assimilated nutrients^49–51^. Interestingly, the *δ*^15^N values of some Menorcan shearwaters, predicted to have remained in the Mediterranean post-breeding, fell below the occupied isotope niche space of sampled fishes, suggesting exploitation of low tropic level prey such as invertebrates^52,53^. Nevertheless, we do not have prey data to test this suggestion further.

The core distributions of shearwaters from the three populations in the October - June period (when birds are engaging in pre-breeding or breeding behaviours) also differed (Fig. 3). Light data suggest that Menorcan birds predominantly occupy waters north and northeast of the colony stretching into the Gulf of Lion, Yelkouan shearwaters centre their distribution on the Gulf of Lion with some use of the Ligurian, Tyrrhenian and Black Seas, and Mallorcan birds occupy areas further west towards Catalan and Valencian coasts. Despite the coarse resolution of geolocator data (186 ± 114 km^54^), this result is consistent with foraging movements of satellite-tracked Menorcan shearwaters during chick-rearing^29^, GPS tracking of incubating Balearic shearwaters from Mallorca^55^, and biogeochemically-inferred partitioning in foraging habitat across Balearic shearwater populations^56^. Our findings indicate that Menorcan birds utilize similar foraging areas to French-breeding Yelkouan shearwaters during both pre-breeding and breeding^35^, demonstrating the potential for population mixing. In contrast, differences in habitat use between Menorcan and other Balearic populations during these periods could potentially promote their divergence or at least reduce further hybridization (e.g.^7^).

In addition to observed population differences in at-sea movements, we found preliminary suggestions of differences in breeding and migratory schedules between Mallorcan, Menorcan and French Yelkouan shearwaters. While limited sample sizes limit the power of statistical comparisons, the timings that birds initiated and ceased daytime activity in the colony, and the laying date, differed between populations, with Mallorcan Balearic shearwaters showing earlier onset than one or both of the other populations. These patterns corroborate earlier casual observations of delayed egg laying and chick fledging by a few weeks in the Menorcan population compared to other Balearic shearwater colonies^30,32^, and suggest that breeding on Menorca may be similar or intermediate to that of Yelkouan shearwaters^57^ (Supplementary Fig. S3 and Table S2). There is evidence that breeding asynchrony in seabirds may have a role in population differentiation^7,58,59^, and it is plausible that offsets in breeding schedules may help to explain the genetic structure of Mediterranean *Puffinus* populations, reducing the rate of natural admixing. Indeed, the Balearic shearwater population on Mallorca has been found to show high degrees of within-population pre-breeding and breeding synchrony^33^. Nevertheless, these preliminary data on phenology suggest that between-population differences are unlikely to be sufficient to stop further natural admixing, and a formal detailed comparison of phenological events is still required.

Observed patterns of at-sea movement and phenology are also consistent with existing information on genetic and morphological attributes of Balearic, Menorcan and Yelkouan shearwaters, providing substantial evidence for broad phenotypic differences between Menorcan birds and the ‘parent’ species, but most notably with Balearic shearwaters. Morphological and osteological differences amongst populations of Balearic shearwaters^20,21,27^, and between Balearic and Yelkouan shearwaters^14,20–22,60,61^ have been well established, with the smaller-sized Menorcan birds showing similar morphologies to Yelkouan populations^21,22,62^. *Puffinus* populations in the Mediterranean also vary in their vocal structure, with Menorcan birds showing intermediate characteristics to the two sibling species, tentatively attributed to body size differences^30,31^. Lastly, population differences in plumage coloration have been observed, with many Menorcan birds (including most tracked birds in the current study) showing white underparts comparable to Yelkouan shearwaters that are rarely observed in Balearic shearwater populations elsewhere in the Balearic archipelago^21^.

## Conclusions

The mechanisms by which divergent movement behaviour arises on the one hand, or influences the genetic structure of populations on the other, are still poorly understood^2,5,63^. However, pre-breeding and post-breeding barriers to gene flow caused by population-specific migratory-related attributes (i.e. breeding schedules or mate choice) have been proposed^1,2,7^. Our findings are consistent with these suggestions, indicating that an interplay between movement behaviour and breeding phenology may play a role in maintaining the genetic structure of contemporary *Puffinus* shearwater populations in the Mediterranean, and could plausibly have been involved in earlier evolutionary divergence of Balearic and Yelkouan shearwaters. Menorcan shearwaters do not fit this divergent pattern, exhibiting spatial, behavioural and genetic traits seen in both Balearic and Yelkouan populations. Our results support recent phenotypic and genetic analyses, indicating that the Menorcan population display an intermediate phenotypic position between the two sibling species. For instance, the use of an intermediate migration strategy to that adopted by Mallorcan and Yelkouan shearwaters could be a behavioural consequence of hybridization in this colony^39,64^, demonstrating that despite the differences between the two parental species admixing can still occur. The fact that Menorcan shearwaters can at least survive and reproduce with their intermediate behavioural strategies, however, suggests that these could simply be explained by the intermediate location of the Menorcan colony in comparison to other *Puffinus* breeding sites in the Mediterranean (Fig. 2). Thus, even if hybridisation is responsible for generating the intermediate phenotype of Menorcan shearwaters, an intermediate ecology at their breeding area might be supportable precisely because of its intermediate geography. Further comparison of the migratory and breeding behaviour of other colonies, including those where birds appear to be less genetically and phenotypically differentiated from those on Menorca^21^, is warranted.

Together, our findings suggest that Menorcan shearwaters represent an important population for the study of evolutionary mechanisms, with potential to provide further insights into processes of speciation and admixing in the Mediterranean. Furthermore, this study identifies a crucial need for conservation strategies that account for inter-population variation in at-sea behaviour, to ensure protection throughout the species’ distribution range. For example, multi-regional efforts at site protection will be required to target important non-breeding areas of all components of the global ‘critically endangered’ Balearic shearwater population fully.

## Methods

### Study site and ethics statement

This study was conducted at two shearwater colonies in the northwestern Mediterranean: 1) Mola de Maó, Menorca, the largest known colony of Menorcan shearwaters – a believed hybrid population (39°52′N, 004°19′E; ~300 pairs^65^) and 2) Sa Cella cave, Mallorca, one of the largest known cave colonies of Balearic shearwaters (39°36′N, 002°21′E; ~200 pairs^65^). Colony work was conducted under permits issued by the Government of the Balearic Islands (permit numbers: CAP31/2011, CEP04/2012, CEP03/2013, CEP15/2014), and following guidelines and established protocols to minimise disturbance (see^33^). To compare newly collected data on Mallorcan and Menorcan shearwater colonies to those on Yelkouan shearwaters, we also reanalysed existing data from the nearest Yelkouan shearwater population in the French Mediterranean (Porquerolles and Port-Cros Islands, Hyeres Archipelago, France; ~381–387 km away from the hybrid colony) following the methods outlined below (full sampling details in^35^). All experimental protocols were approved by the National Oceanography Centre, Southampton and/or the Balearic Islands Government.

### Bio-logging

Between March 2011 and April 2014, the year-round movements of birds from both Mallorcan and Menorcan colonies were tracked using BAS geolocators (British Antarctic Survey, Cambridge, UK; Models: MK15, MK18, and MK19; Weight: 1.9–2.5 g; 0.50 ± 0.07% and 0.43 ± 0.08% of body mass for Menorcan and Mallorcan birds, respectively). During the breeding season, between March and May, devices were attached to the tarsus of birds using a plastic ring, and recovered during the subsequent year. Ringed individuals were either removed directly from the nest while incubating (both colonies), or captured in cave entrances prior to pair switchovers where nests were inaccessible (Menorca). Birds were handled for a mean duration of 19.7 (±SD 7.7) minutes on Menorca and 22.2 (±SD 8.9) minutes on Mallorca.

Geolocation light data were processed with BAStrak software (British Antarctic Survey, Cambridge, UK), using a light threshold of 10. Light curves were manually assessed and discarded in the presence of obvious light interference during sunrise and sunset transitions. Data were filtered to remove dark periods of <4 hrs, unreliable fixes around the equinoxes (10 days on the winter side, and 5 days on the summer side), unrealistic locations >52° and <30°N, and locations associated with unrealistic travel distances based on a maximum travel speed of 55 km h^−1^ ^55^. The sensitivity of individual devices to different sun elevation angles (EA) was calibrated during the late incubation and chick-rearing period, when it was assumed that the birds’ movements were centred near the colony. The angle that resulted in the smallest locational bias, and/or fewest points on land, during this period was used for each geolocator (EA: −3.0° to −4.0°).

Balearic shearwaters predominantly attend colonies between October and June, but do not begin incubating until the early spring^33^. To investigate phenology of the focal populations, we defined the start and end dates of the non-breeding period as the birds’ last and first night visits to the colony, respectively. Following^33^, night visits were identified as periods of continuous night-time dryness in the geolocator salt-water immersion trace (≥2 hrs), while day time visits to the colony were identified as complete daytime darkness in the light trace. The median first night visit for birds with a full year of recovered data was used as the end of the non-breeding period for birds whose activity loggers had failed prior to their return to the colony. Both activity and light data were used to estimate laying date, based on patterns of asynchronous daytime visits to the colony between pair members (or when only one pair member was tracked, the onset of regular daytime visits lasting ≥2 days in males, and first colony visit after the post-laying exodus in females). As events later in the breeding cycle (i.e. migration leaving date) are susceptible to breeding failure (which was not measurable for most birds from Menorca and France due to accessibility constraints), we compared only the first cave day visit, laying date and last cave day visit (estimated hatch date), which can be inferred consistently.

The core non-breeding areas of individual birds from Menorca were determined using fixed kernel density estimation (KDE) in the ‘KernSmooth’ package in R^66^ (data projection: lambert conformal conic; cell size: 1 × 1 km). Optimised KDE bandwidths were obtained for each bird using the multivariate plug-in selector in the ‘ks’ package in R^67,68^. Seasonal population-level KDEs (3-monthly intervals) were also obtained for the three populations for periods with sufficient comparable data (June, July-September, October-December, January-March). Partial data collected during April and May were excluded here as these months coincided with logger deployment and recovery periods at some colonies, and thus contained periods of missing data and small sample sizes.

### Stable isotopes

The carbon and nitrogen stable isotope compositions of animal tissues reflect diet during tissue growth. Isotope compositions of individuals moving between isotopically distinct habitats can therefore provide information on the spatial location of feeding^46^. To aid investigation of non-breeding movements, and support interpretation of the limited spatial data from Menorcan shearwaters, feather stable isotope data were collected from a larger number of birds at focal colonies. For the Mallorcan and Menorcan populations, primary flight feathers representing the early (P1) and central (P6) parts of the non-breeding season^38^ were sampled from both tracked and untracked birds during spring 2012 and 2013 (Tracked birds, Menorca: *n* = 4, Mallorca: *n* = 32; Untracked birds, Menorca: *n* = 32, Mallorca: *n* = 9; feathers representative of diet during the 2011 and 2012 non-breeding periods). Feather choice was based on extensive moult scoring of Balearic shearwaters from at-sea images and at colonies (see^69^). To obtain reference isotope data for the western Mediterranean region, feather down from 40 Mallorcan chicks was sampled in June 2013. Feathers were washed in a solution of 0.25 M sodium hydroxide, rinsed in successive washes of milli-Q water and oven dried at 50 °C until reaching constant mass. Samples were then cut into ~1 mm pieces prior to weighing. We then compared data from Balearic shearwaters to available temporally matching isotope values for Yelkouan shearwaters sampled from P1 feathers grown during 2011^35^ (*n* = 33).

Stable isotope analyses (SIA) of Balearic shearwater samples were carried out at the Natural Environment Research Council Life Science Mass Spectrometry Facility in East Kilbride, using a Flash HT elemental analyser (2012) or Elementar vario PYRO cube elemental analyser (2013) coupled with a Thermo Electro Delta XP continuous flow isotope ratio mass spectrometer. Isotope ratios were expressed in delta notation in parts per thousand (‰), relative to international standards of *δ*^13^C (Pee Dee Belemnite, V-PCB) and *δ*^15^N (atmospheric N_2_, AIR). Multiple measurements of three internal laboratory standards (gelatine, alanine and glycine) in each SIA experiment indicated that measurement error was smaller than 0.2‰ for *δ*^15^N and 0.1‰ for *δ*^13^C.

We used hierarchical clustering analysis with the Ward’s minimum variance method on stable isotope data from P1 and P6 feathers (separately), to investigate the degree to which Balearic and Yelkouan shearwaters that used different non-breeding areas clustered together isotopically. To describe the distribution of stable isotope measurements in two-dimensional isotope space, standard ellipse areas (SEAc: corrected for smaller sample sizes), Bayesian approximations of standard ellipse areas (SEAb) and convex hulls were calculated for groups obtained via hierarchical clustering using the SIAR package in R^70,71^. Discriminant function analysis (DFA) was then performed on P1 (Balearic and Yelkouan shearwaters) and P6 (Balearic shearwaters) isotope values, representative of the beginning and central parts of the non-breeding season^38^, to examine how well isotopic tracers discriminated between the non-breeding regions of birds, and allow predictions to be made for untracked birds. The isotope values of 38 tracked individuals for P1 (Mediterranean = 19, Atlantic = 19; no repeated individual measures) and 26 tracked individuals for P6 (Mediterranean = 8, Atlantic = 18; no repeated individual measures) were first used as training datasets to develop the discriminant functions, before predictions were made for 62 and 41 untracked birds for P1 and P6, respectively (P1 = 9 Mallorcan, 32 Menorcan, 21 Yelkouan; P6 = 32 Menorcan, 9 Mallorcan). The obtained DFAs were validated using a jackknife leave-one-out cross-validation method on training data.

In order to disentangle spatial and trophic influences on feather stable isotope compositions from the Mediterranean and Atlantic, isotopic ratios of P6 feathers from Mallorcan and Menorcan shearwaters, grown during the non-breeding season in 2012, were compared to fractionation-corrected isotope ratios in pelagic and demersal fish muscle samples (see Supplementary Table S1 for sample details). P1 isotope values from Yelkouan shearwaters grown in 2011 (the nearest temporal match to sampled prey) were also presented as a comparison. As there are no published feather-prey muscle isotopic discrimination factors for adult Procellariiformes, the means of published discrimination factors for similar seabird species were applied to prey data (*δ*^15^N = 3.7‰, *δ*^13^C = 1.9‰; see^38^ for details). Fish samples were collected from coastal fisheries operating out of Vilanova i la Geltrú, Spain during May 2012, and both Lorient, France and Aveiro, Portugal during July 2012. Fish muscle *δ*^13^C values for samples with C:N ratios >3.15 were lipid-corrected using lipid-normalization equations from^72^, and analysed following the methods outlined above (details in^38^).

## Supplementary information

Supplementary Information

## Data Availability

The datasets generated during and/or analysed during this study are available from the corresponding author on reasonable request.
