# Supplementary material for: Patterns of at-sea behaviour at a hybrid zone between two threatened seabirds
Source: Sci Rep. 2019 Oct 11;9:14720. doi: 10.1038/s41598-019-51188-8 (PMC6789130; doi:10.1038/s41598-019-51188-8)
Supplement: Supplementary file 1 — Supplementary Information [file 41598_2019_51188_MOESM1_ESM.pdf]

The following supplementary information accompanies the article:

Patterns of at-sea behaviour at a hybrid zone between two threatened seabirds

Rhiannon E. Austin[*1,#a], Russell B. Wynn[1], Stephen C. Votier[2], Clive Trueman[3], Miguel McMinn[4], Ana Rodríguez[4], Lavinia Suberg[1], Louise Maurice[5], Jason Newton[6], Meritxell Genovart[7,8], Clara Péron[9,10], David Grémillet[9,11], Tim Guilford[12]

[1]National Oceanography Centre - Southampton, European Way, Southampton, SO14 3ZH, UK.
[2]Environment and Sustainability Institute, University of Exeter, Penryn Campus, Penryn, Cornwall, TR10 9FE, UK
[3]Ocean and Earth Sciences, University of Southampton Waterfront Campus, Southampton, SO14 3ZH, UK.
[4]Grupo Biogeografía, geodinámica y sedimentación del Mediterráneo occidental (BIOGEOMED), Universitat de les Illes Balears, Cra. de Valledemossa, km 7.5 E07122 Palma, Balearic Islands, Spain
[5]British Geological Survey, Natural Environment Research Council, Benson Lane, Crowmarsh Gifford, Oxfordshire, OX10 8BB, UK
[6]NERC Life Sciences Mass Spectrometry Facility, Scottish Universities Environmental Research Centre, East Kilbride, Scotland, G75 0QF, UK
[7]IMEDEA (CSIC-UIB), Miquel Marquès 21, 07190 Esporles, Mallorca, Spain
[8]CEAB (CSIC), Accés Cala Sant Francesc 14, 17300 Blanes, Girona, Catalonia, Spain
[9]Centre d'Ecologie Fonctionnelle et Evolutive, UMR 5175, CNRS – Université de Montpellier - Université Paul-Valéry Montpellier - EPHE, Montpellier, France
[10]Muséum national d'Histoire naturelle, Département Adaptations du vivant, UMR 7208 BOREA (MNHN, CNRS, IRD, Sorbonne Université, UCB, UA), CP 26, 43 rue Cuvier, 75231 Paris cedex 05, France
[11]FitzPatrick Institute, DST-NRF Centre of Excellence at the University of Cape Town, Rondebosch 7701, South Africa
[12]Department of Zoology, University of Oxford, 11a Mansfield Road, Oxford, OX1 3SZ, UK
[#a]Current Address: School of Environmental Sciences, University of Liverpool, Nicholson Building, Brownlow Street, Liverpool, L69 3GP, UK

*Corresponding author: Rhiannon E. Austin
Email: rhiannoneaustin@gmail.com

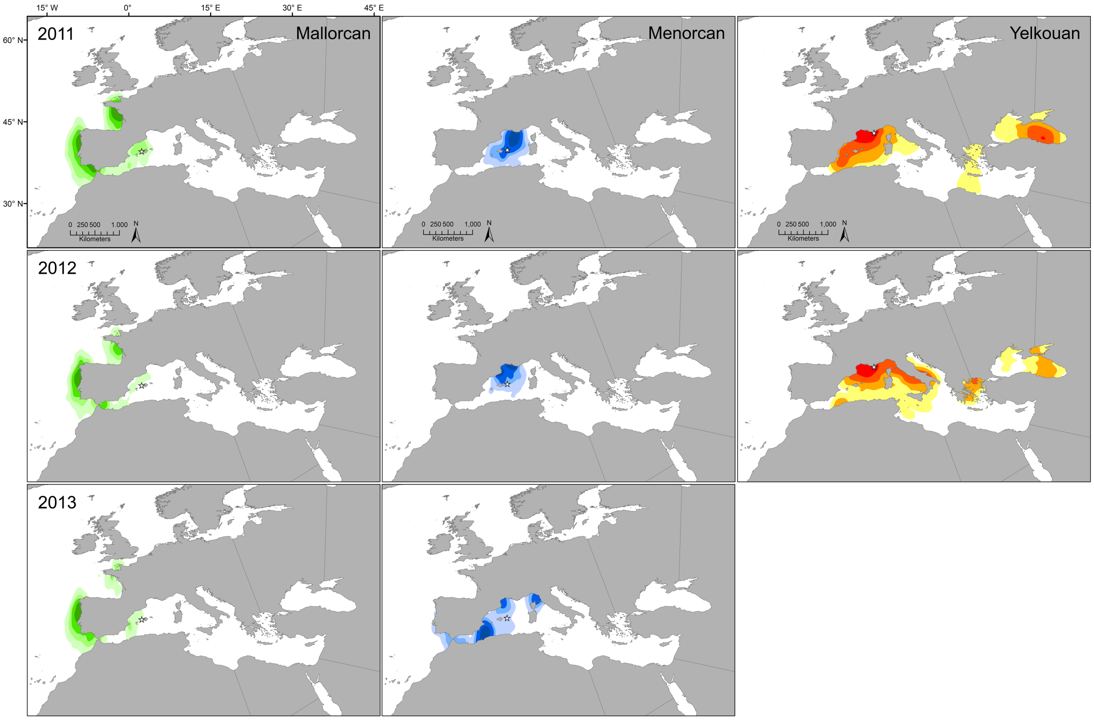

Supplementary Figure S1. The non-breeding distributions of Mallorcan, Menorcan and Yelkouan shearwater populations by year. Kernel density estimates of shearwaters tracked with geolocators from Sa Cella, Mallorca (*n*, 2011 = 16, 2012 = 16, 2013 = 20), La Mola, Menorca and the Hyéres Archipelago in the French Mediterranean (*n*, 2011 = 15, 2012 = 19) are shown. Bandwidth selector = plug-in; 25%, 50%, 70% and 90% kernel density contours are given. Maps created in ArcGIS version 10.3 (ESRI, USA; http://desktop.arcgis.com/en/arcmap/).

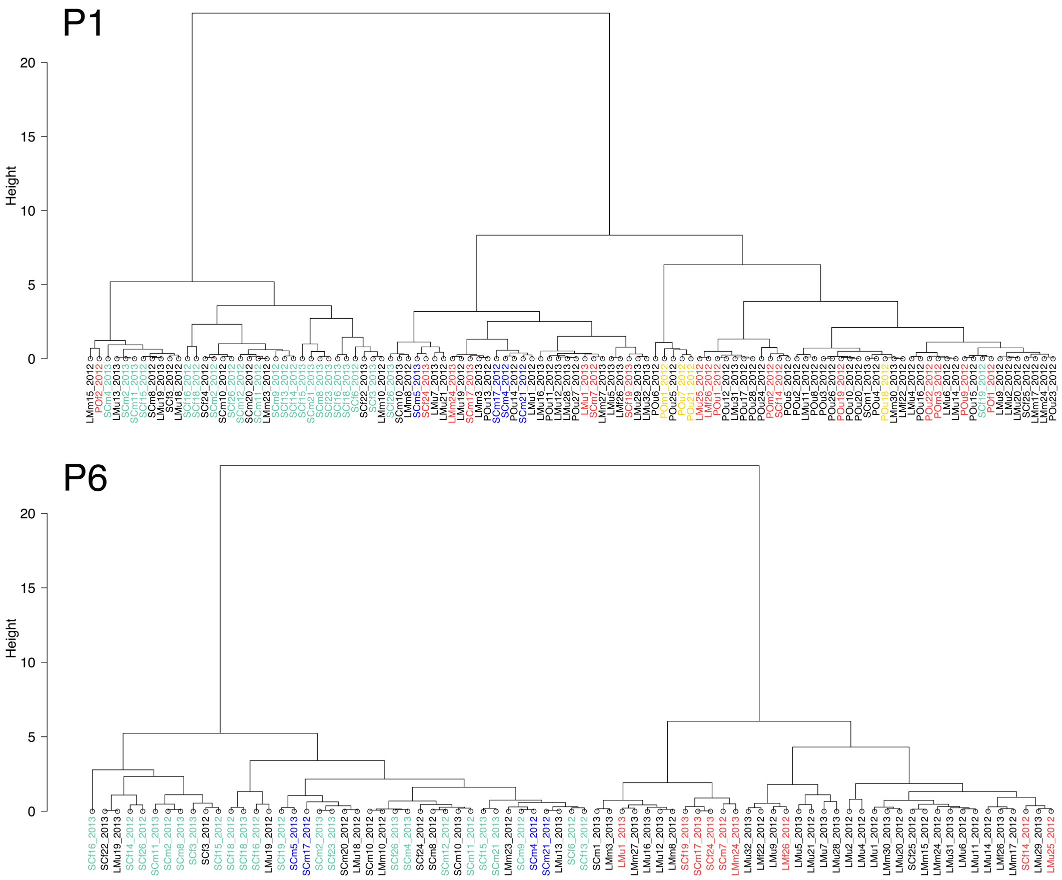

Supplementary Figure S2. Dendrograms of the isotopic similarity between Mallorcan, Menorcan and Yelkouan shearwater feathers. Dendrograms show the isotopic similarity between samples according to hierarchical clustering analysis with Wards' minimum variance method on δ^{15}N and δ^{13}C data for a) P1 feathers from Mallorcan (SC) and Menorcan (LM) shearwaters sampled during 2012 and 2013, and Yelkouan shearwaters from Port-Cros, France (PO) sampled during 2012[1] and b) P6 feathers from the Mallorcan and Menorcan colonies only. Label colours indicate the oceanic region where birds spent the non-breeding season: green = Atlantic, red = Mediterranean, blue = both regions, gold = Black Sea, black = unknown. m = males; f = females; u = unknown sex.

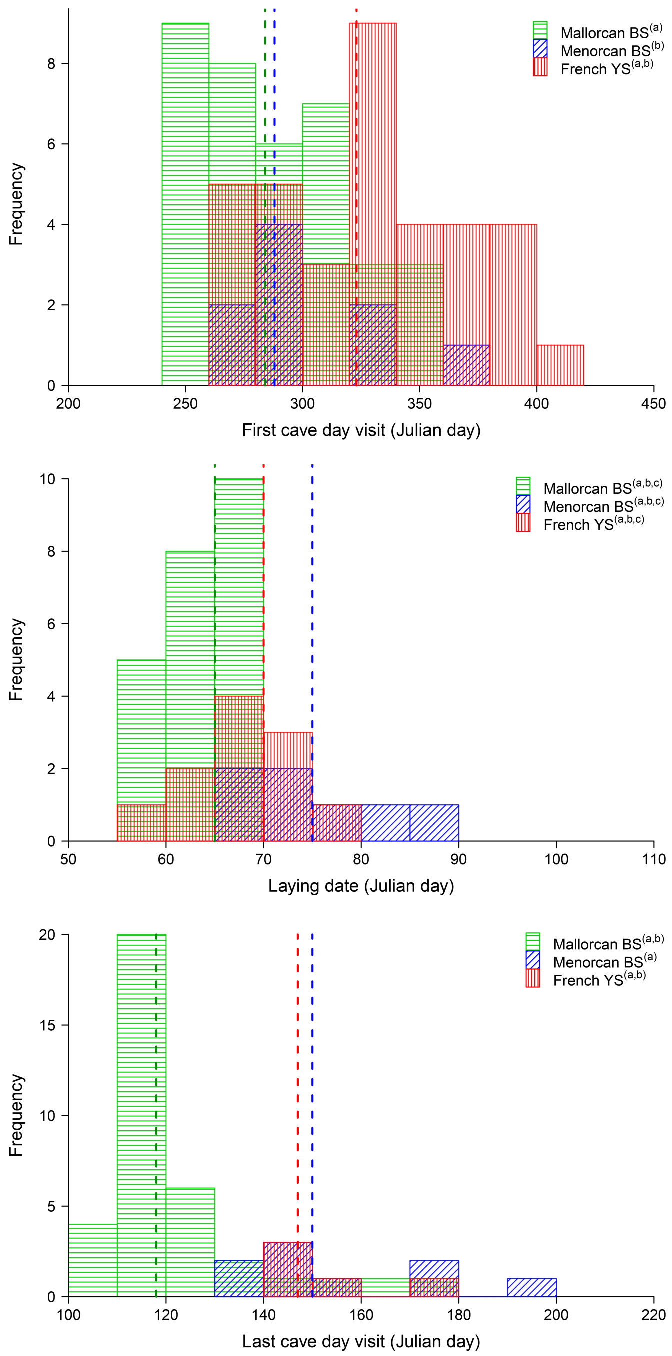

Supplementary Figure S3. Breeding phenology of Mallorcan, Menorcan and Yelkouan shearwaters. The first and last day visits to the colony, and estimated laying dates of GLS-tracked Balearic shearwaters from Mallorca, Menorcan shearwaters from La Mola, Menorca, and Yelkouan shearwaters from the Hyères Archipelago, France, between 2013 and 2014. Known failed breeders from the Mallorcan colony (where breeding failure was detectable) are not plotted. Dotted lines show the median Julian day of each respective event for the three colonies. Superscript letters show results of tukey pairwise comparisons from LMMs, with colonies that share the same letter showing significant differences. As Julian day values are circular and, within a tracking period, 'first day visits to the colony' ranged between September of the first year and February of the second year, cumulative Julian day values were used for statistical modelling, with days from 1st January in the second year starting at 366.

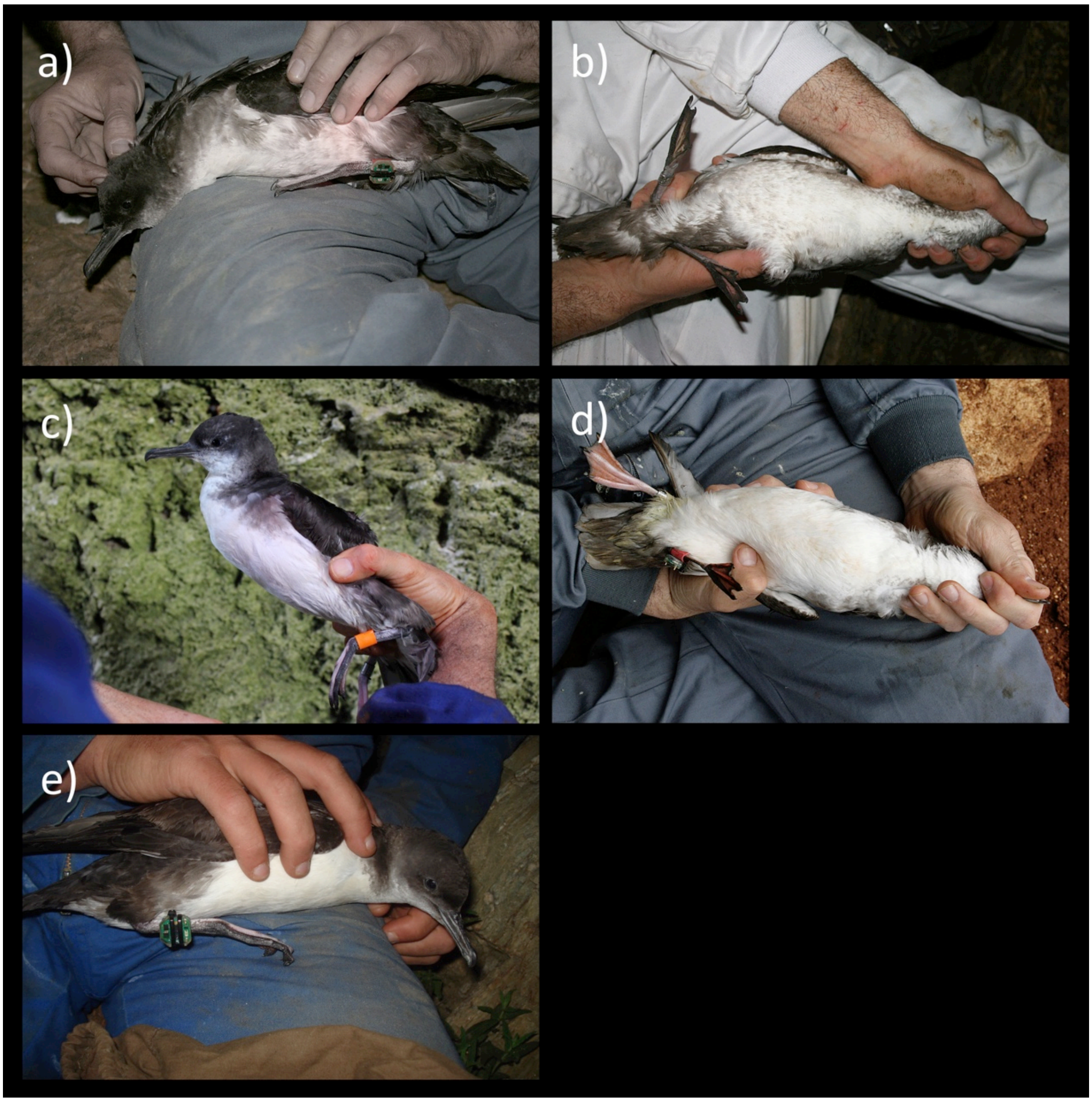

Supplementary Figure S4. Images of Balearic shearwaters from Mallorca (a,b), Menorcan shearwaters from La Mola, Menorca (c,d), and a Yelkouan shearwater from the Hyères Archipelago, French Mediterranean (e). Both Mallorcan birds (a,b) show intermediate Balearic shearwater colouration, while both Menorcan shearwaters (c,d) show pale yelkouan-type colouration. Moult score: a,b = 3 - 4, c = 2, d = 1, e = 1 (scale 1 - 5, where 1 = yelkouan-type with pure white underparts, mostly white undertail feathers and an incomplete collar, and 5 = dark Balearic-type with dark underparts and a nearly complete collar; see [2]). Photographs: a-d = R.B. Wynn (2011-2013), e = K. Bourgeois (2013).

Supplementary Table S1. Stable isotope values of potential prey samples. Mean (± SD) muscle carbon and nitrogen stable isotope values, and total lengths of fish samples from the northeast Atlantic and western Mediterranean regions used in the study. Atlantic samples were collected in July 2012 from fisheries operating in the Bay of Biscay and off western Iberian coasts. Mediterranean samples were collected in May 2012 from fisheries operating off the Catalan coast.

	NE. Atlantic				W. Mediterranean			
Species	*n*	δ^{15}N	δ^{13}C	TL	*n*	δ^{15}N	δ^{13}C	TL
Sardina pilchardus (P)	20	11.3 (±0.6)	-17.8 (±0.5)	14.2 (±2.6)	10	8.4 (±0.4)	-19.4 (±0.2)	12.4 (±0.2)
Engraulis encrasicolus (P)	14	11.6 (±0.7)	-17.6 (±0.4)	14.3 (±0.9)	10	8.0 (±0.3)	-19.1 (±0.1)	11.1 (±0.5)
Sardinella aurita.(P)	-	-	-	-	10	8.2 (±0.3)	-19.4 (±0.2)	14.4 (±0.6)
Trachurus trachurus (P)	10	11.6 (±0.9)	-18.8 (±0.6)	11.5 (±1.0)	-	-	-	-
Micromesistius poutassou (P)	-	-	-	-	10	7.7 (±0.3)	-19.0 (±0.1)	16.8 (±0.5)
Merluccius merluccius (P)*	10	10.2 (±0.4)	-19.6 (±0.2)	15.4 (±1.1)	-	-	-	-
Trisopterus luscus (D)	15	12.4 (±0.8)	-17.7 (±1.0)	15.4 (±3.9)	-	-	-	-
Dicologlossa cuneata (D)	10	12.1 (±0.5)	-16.7 (±0.3)	15.1 (±0.9)	-	-	-	-
Chelidonichthys lucerna (D)	10	13.4 (±0.5)	-16.4 (±0.5)	15.4 (±1.8)	-	-	-	-
Phycis blennoides (D)	-	-	-	-	5	9.7 (±0.4)	-19.1 (±0.1)	18.8 (±1.3)
Lampanyctus crocodilus (D)	-	-	-	-	10	7.2 (±0.4)	-18.8 (±0.2)	11.1 (±0.8)
Boops boops (D)	-	-	-	-	10	9.2 (±0.3)	-19.3 (±0.2)	14.7 (±1.2)
Spicara maena (D)	-	-	-	-	4	10.2 (±0.5)	-18.3 (±0.6)	18.4 (±0.5)

P = pelagic; D = demersal; TL = total length.

*Juvenile *M. merluccius* (≤16.5 cm TL) grouped with pelagic species owing to the likely diet of this size class (see [3]).

Supplementary Table S2. Summary of migration and breeding phenology of geolocator-tracked Mallorcan, Menorcan and Yelkouan shearwaters between 2011 and 2014. Mean (± SD) values for Balearic shearwaters from Mallorca, Menorcan shearwaters from Menorca and Yelkouan shearwaters from the Hyères archipelago in the French Mediterranean [35] are given. The last day and night visits to the colony during the first breeding season, and the first night and day visits to the colony during the second breeding season are shown. Numbers in brackets represent overall sample sizes. Values for all tracked birds, as well as for only known successful breeders (SBs), are shown for the Mallorcan population. NB = non-breeding. *n* for lay date: Mallorca – *SBs*, 2013-2014 = 7, 2012-2013 = 7, 2011-2012 = 9; Menorca, 2013-2014 = 4, 2012-2013 = 1, 2011-2012 = 2; France, 2011-2012 = 11. Combined means ± SD values (all years) are given in Julian days.

Bird	Year	Last cave day (season 1)	Last cave night (season 1)	First cave night (season 2)	First cave day (season 2)	Estimated lay date	NB duration (days)
Mallorca (20)<br>Mallorca (15) - SBs	2013-2014<br>2013-2014	08/05/2013 ±18<br>04/05/2013 ±19	06/06/2013 ±15<br>11/06/2013 ±12	20/09/2013 ±18<br>19/09/2013 ±19	25/09/2013 ±21<br>25/09/2013 ±21	06/03/2014 ±2<br>04/03/2013 ±2	106 ±19<br>101 ±18
Mallorca (16)<br>Mallorca (12) - SBs	2012-2013<br>2012-2013	06/05/2012 ±15<br>05/05/2012 ±19	14/06/2012 ±23<br>26/06/2012 ±7	02/10/2012 ±22<br>10/10/2012 ±23	05/10/2012 ±22<br>14/10/2012 ±22	02/03/2013 ±4<br>01/03/2012 ±4	110 ±23<br>103 ±25
Mallorca (16)<br>Mallorca (12) - SBs	2011-2012<br>2011-2012	27/04/2011 ±8<br>24/04/2011 ±5	12/06/2011 ±18<br>19/06/2011 ±12	15/10/2011 ±25<br>23/10/2011 ±22	27/10/2011 ±37<br>07/11/2011 ±33	08/03/2012 ±2<br>08/03/2011 ±2	124 ±18<br>126 ±14
Menorca (6)	2013-2014	20/06/2013 ±17	06/07/2013 ±8	20/10/2013 ±20	04/11/2013 ±29	13/03/2014 ±7	104 ±25
Menorca (2)	2012-2013	27/05/2012 ±4	27/05/2012 ±3	03/10/2012 ±16	04/10/2012 ±16	17/03/2013	128 ±18
Menorca (2)	2011-2012	14/05/2011 ±1	07/06/2011 ±31	13/09/2011 ±13	13/11/2011 ±71	23/03/2012 ±8	96 ±45
France, YS (20)	2012-2013	03/06/2012 ±20	27/06/2012 ±17	03/11/2012 ±32	16/11/2012 ±41	-	123 ±29
France, YS (17)	2011-2012	02/06/2011 ±8	22/06/2011 ±17	17/10/2011 ±31	12/12/2011 ±34	10/03/2012 ±6	116 ±34
**Mallorca (SBs-All years)**	-	**122 ±16 (01/05) [36]**	**169 ±14 (18/06) [36]**	**279 ±25 (06/10) [36]**	**288 ±31 (15/10) [36]**	**64 ±4 (05/03) [23]**	**110 ±21 [36]**
**Menorca (All years)**	-	**157 ±21 (06/06) [9]**	**173 ±22 (22/06) [10]**	**282 ±23 (09/10) [10]**	**303 ±36 (30/10) [9]**	**75 ±6 (16/03) [7]**	**108 ±28 [9]**
**France (All years)**	-	**154 ±15 (03/06) [5]**	**176 ±17 (25/06) [35]**	**301 ±32 (28/10) [28]**	**332 ±40 (28/11) [35]**	**69 ±6 (10/03) [11]**	**120 ±31 [27]**

References

1 Péron, C. *et al*. Importance of coastal Marine Protected Areas for the conservation of pelagic seabirds: The case of vulnerable Yelkouan shearwaters in the Mediterranean Sea. *Biol. Conserv*. **168**, 210-221 (2013).

2 Genovart, M., Juste, J., Contreras-Díaz, H. & Oro, D. Genetic and phenotypic differentiation between the critically endangered Balearic shearwater and neighboring colonies of its sibling species. *J. Hered.* **103**, 330-341 (2012).

3 Ferraton, F., Harmelin Vivien, M., Mellon-Duval, C. & Souplet, A. Spatio-temporal variation in diet may affect condition and abundance of juvenile European hake in the Gulf of Lions (NW Mediterranean). *Mar. Ecol. Prog. Ser.* **336**, 197-208 (2007).
